# High serum sCD163/sTWEAK ratio is associated with lower risk of digital ulcers but more severe skin disease in patients with systemic sclerosis

**DOI:** 10.1186/ar4246

**Published:** 2013-06-24

**Authors:** Otylia Kowal-Bielecka, Marek Bielecki, Serena Guiducci, Beata Trzcinska-Butkiewicz, Małgorzata Michalska-Jakubus, Marco Matucci-Cerinic, Marek Brzosko, Dorota Krasowska, Lech Chyczewski, Krzysztof Kowal

**Affiliations:** 1 Department of Rheumatology and Internal Medicine, Medical University of Bialystok, Bialystok, Poland; 2 Department of Orthopedics and Traumatology, Medical University of Bialystok, Bialystok, Poland; 3 Department of BioMedicine, Division of Rheumatology AOUC, & Department of Medicine, Denothe Center, University of Florence, Florence, Italy; 4 Department of Rheumatology and Internal Medicine, Medical University of Szczecin, Szczecin, Poland; 5 Department of Dermatology, Venereology and Pediatric Dermatology, Medical University of Lublin, Lublin, Poland; 6 Department of Medical Pathomorphology, Medical University of Bialystok, Bialystok, Poland; 7 Department of Allergology and Internal Medicine, Medical University of Bialystok, Bialystok, Poland

## Abstract

**Introduction:**

Systemic sclerosis (SSc) is an autoimmune disease characterized by chronic inflammation, vascular injury and excessive fibrosis. CD163 is a scavenger receptor which affects inflammatory response and may contribute to connective tissue remodelling. It has recently been demonstrated that CD163 can bind and neutralize the TNF-like weak inducer of apoptosis (TWEAK), a multifunctional cytokine which regulates inflammation, angiogenesis and tissue remodelling. We aimed to investigate the relationships between serum levels of soluble CD163 (sCD163) and soluble TWEAK (sTWEAK) in relation to disease manifestations in SSc patients.

**Methods:**

This study included 89 patients with SSc who had not received immunosuppressive drugs or steroids for at least 6 months and 48 age- and sex-matched healthy controls (HC) from four European centres. Serum concentrations of sTWEAK and sCD163 were measured using commercially available ELISA kits.

**Results:**

The mean serum concentrations of sTWEAK were comparable between SSc patients (mean +/- SD: 270 +/- 171 pg/mL) and HC (294 +/- 147pg/mL, *P *>0.05). Concentration of sCD163 and sCD163/sTWEAK ratio were significantly greater in SSc patients (984 +/- 420 ng/mL and 4837 +/- 3103, respectively) as compared to HC (823 +/- 331 ng/mL and 3115 +/- 1346 respectively, *P *<0.05 for both). High sCD163 levels and a high sCD163/sTWEAK ratio (defined as > mean +2SD of HC) were both associated with a lower risk of digital ulcers in SSc patients (OR, 95%CI: 0.09; 0.01, 0.71, and 0.17; 0.06, 0.51, respectively). Accordingly, patients without digital ulcers had a significantly higher sCD163 concentration and sCD163/sTWEAK ratio as compared to SSc patients with digital ulcers (*P *<0.01 for both) and HC (*P *<0.05 for both). A high sCD163/sTWEAK ratio, but not high sCD163 levels, was associated with greater skin involvement.

**Conclusions:**

The results of our study indicate that CD163-TWEAK interactions might play a role in the pathogenesis of SSc and that CD163 may protect against the development of digital ulcers in SSc. Further studies are required to reveal whether targeting of the CD163-TWEAK pathway might be a potential strategy for treating vascular disease and/or skin fibrosis in SSc.

## Introduction

Systemic sclerosis (SSc, scleroderma) is a chronic, autoimmune disease, which affects skin, the lungs, heart, gastrointestinal tract and the kidneys. Excessive production of extracellular matrix by activated fibroblasts and widespread vascular injury are believed to play a key role in the pathogenesis of SSc. In addition, inflammatory infiltrates consisting mainly of mononuclear cells are found in the skin and affected organs, in particular in the early stage of the disease [1,2].

CD163 is a member of the scavenger receptor cysteine-rich (SRCR) family, which is expressed exclusively in monocytes/macrophages and takes part in the uptake of circulating haptoglobin-haemoglobin complexes [3,4]. Shedding of membrane CD163 through a proteolytic process produces soluble CD163 molecule (sCD163), which can be detected in body fluids [5-7]. Both membrane bound and soluble CD163 (sCD163) exert strong anti-inflammatory effects [8]. Uptake of haptoglobin-haemoglobin complexes by CD163-expressing macrophages leads to increase in production of interleukin-10 and expression of haeme oxygenase -1 [9]. Soluble CD163 can directly inhibit T cells proliferation [10,11]. Expression of CD163 on tissue macrophages increases during the resolution phase of acute inflammatory response [8]. Similarly, increase in plasma levels of sCD163 and expression of CD163 on monocytes have been associated with lack of prolonged airway inflammation after allergen challenge in asthmatic patients [12]. Moreover, expression of CD163 increases during the wound healing phase, indicating a possible role of CD163 in the regulation of connective tissue remodelling [13,14].

Recently, it has been shown that CD163 can specifically bind and neutralize TNF-like weak inducer of apoptosis (TWEAK), a newly identified pleiotropic cytokine belonging to the TNF-α superfamily of cytokines [15]. TWEAK is involved in the regulation of inflammation, angiogenesis and tissue repair and remodelling [16,17]. Interacting with its specific receptor, fibroblast growth factor-inducible 14 (Fn14) (TWEAKR), TWEAK activates NF-kB and mitogen-activated protein (MAP) kinase pathways and induces production of pro-inflammatory mediators, including interleukin-6, interleukin-8, monocyte chemotactic protein-1 and several metalloproteinases by different cell types, including macrophages, fibroblasts, and endothelial cells [17-21]. TWEAK also stimulates endothelial cell migration, proliferation and survival and cooperates with other pro-angiogenic factors such as vascular endothelial growth factor (VEGF) and fibroblast growth factor (FGF) in the regulation of development of new blood vessels [16]. Moreover, TWEAK can directly activate fibroblasts [19-21].

As mentioned above, binding of TWEAK to CD163 neutralizes TWEAK and in this way inhibits its function [15]. Moreover, it has recently been shown that CD163-expressing monocytes/macrophages are able to bind and internalize exogenous TWEAK [22]. It has also been shown that sCD163/sTWEAK ratio in peripheral blood is a more sensitive biomarker of subclinical atherosclerosis than sCD163 or sTWEAK alone, indicating that interactions between TWEAK and CD163 might play an important role *in vivo *[22]. Since both CD163 and TWEAK have been implicated in the regulation of fibroblast function, their interactions might also be of importance for connective tissue remodelling.

Only a few papers have reported on the evaluation of serum levels of sCD163 or sTWEAK in SSc. However, none have addressed the issue of the mutual relationship between sCD163 and sTWEAK in SSc [23-25]. Taking into account molecular interaction between TWEAK and CD163 molecules, in the present study we evaluated concentration of both proteins in a sample of 89 SSc patients and 48 healthy controls.

## Patients and methods

Eighty-nine patients and appropriate healthy controls (HC) from four clinical centres were enrolled. All patients fulfilled the American College of Rheumatology (ACR) classification criteria for SSc and/or Medsger and LeRoy criteria for early SSc [26,27]. Only patients who had not received immunosuppressive therapies or steroids for at least 6 months before the study were considered eligible. In addition, all patients with known coronary artery disease, clinical manifestations of atherosclerosis, such as stroke or peripheral obliterative arterial disease, as well as those with symptoms of infection were excluded from the study. Patients were classified as having diffuse cutaneous SSc (dSSc) or limited cutaneous SSc (lSSc) as defined by LeRoy and Medsger [28]. Patients' assessment included evaluation of duration of Raynaud's phenomenon and duration of the disease defined from the first non-Raynaud's symptom attributable to SSc. In order to better understand the relationship between disease duration and changes in expression of investigated molecules, patients were divided into those with short- and long-term disease. In agreement with previous studies, patients were considered as having short-term disease if disease duration was not longer than 3 years in dSSc or 5 years in lSSc measured from the first non-Raynaud symptom [29-31]. Accordingly, patients with dSSc in whom disease lasted longer than 3 years and patients with lSSc in whom disease lasted longer than 5 years were considered as having long-term disease.

The extent of skin involvement was assessed using the modified Rodnan skin score (mRSS), as described elsewhere [32]. The presence of scleroderma interstitial lung disease (ILD) was defined based on the typical findings in high resolution computed tomography (HRCT) of the lungs. Pulmonary function tests were used for assessment of the severity of lung involvement and included measurements of forced vital capacity (FVC) and diffusion capacity of the lungs for carbon monoxide (DLCO). Pulmonary artery systolic pressure (PASP) was calculated based on the tricuspid regurgitation velocity on Doppler echocardiography. The presence of pulmonary hypertension (PH) was defined as PASP higher than 45 mmHg, which was shown to have 95% specificity versus right heart catheterization, which is considered a gold standard for diagnosis of PH [33]. Scleroderma renal crisis was defined as new renal insufficiency with or without arterial hypertension, which could not be explained other than by SSc.

As in previous studies, digital ulcers (DU) were defined as a painful area of loss of tissue located on the volar surface of the fingertips or around the nail distal to the proximal interphalangeal digital crease, present at the time of blood collection [34].

The presence of antinuclear antibodies (ANA) and anticentromere antibodies (ACA) was assessed by indirect immunofluorescence and the presence of anti-topoisomerase antibodies (anti-topo I) was evaluated using ELISA. Erythrocyte sedimentation rate (ESR) was used as a laboratory parameter of activity of systemic inflammation.

The control group consisted of 48 age- and sex-matched healthy subjects without evidence of any disease including atherosclerosis or infection (Table 1).

**Table 1:** Clinical characteristics of the patients with systemic sclerosis and the control group.

Parameter^a^	SSc patients (*n *= 89)	Controls (*n *= 48)
Female/male ratio	80/9	42/6^c^
Age, years	57.23 ± 12.31	53.71 ± 14.38^c^
Disease duration, years^b^	10.65 ± 9.00	
Duration of Raynaud's phenomenon, years (data available for 54 patients only)	9.59 ± 8.45	
dSSc/lSSc, number of patients	20/69	
ANA-positive, n (%) patients	84 (94.38)	
Anti-topo I-positive, n (%) patients	35 (39.33)	
ACA-positive, n (%) patients	33 (37.08)	
ILD by HRCT/radiograph, n (%) patients	55 (61.78)	
Pulmonary hypertension, n (%) patients (data available for 81 patients only)	4 (4.94)	
Digital ulcers, n (%) patients	38 (42.70)	
Scleroderma renal crisis, n (%) patients	0	
Arterial hypertension, n (%) patients	8 (9.00)	0
CCBs, n (%) patients Pentoxyphylline, n (%) patients ACEIs, n (%) patients Sildenafil, n (%) patients NSAIDs, n (%) patients	27 (30.34) 33 (37.08) 12 (13.48) 4 (4.49) 13 (14.61)	0 0 0 0 0

Values are given as mean ± SD unless stated otherwise. ^a^Data available for 89 patients unless stated otherwise; ^b^calculated from the time of the first non-Raynaud's symptom attributable to SSc; ^c^statistically non-significant (*P *>0.05). ACA, anticentromere antibodies; ACEIs, angiotensin-converting enzyme inhibitors; ANA, antinuclear antibodies; CCBs, calcium channel blockers; dSSc, diffuse systemic sclerosis; HRCT, high resolution computed tomography; lSSc, limited systemic sclerosis; ILD, scleroderma interstitial lung disease; NSAIDs, non-steroidal anti-inflammatory drugs. Detailed definitions of particular organ involvement are given in the methods section.

The study protocol was approved by the bioethics committee at the Medical University of Bialystok, and informed consent was obtained from all patients.

### Measurements of sCD163 and sTWEAK in the sera

Peripheral blood was collected from cubical vein after the patient had fasted for at least 10 hours; the blood was allowed to coagulate for 30 minutes. Subsequently, samples were centrifuged, and sera were collected and stored at -80°C until measurements were taken [35]. Serum sCD163 and sTWEAK levels were measured using commercially available ELISA kits (sCD163 from R&D Systems, Inc. Minneapolis, MN, USA; sTWEAK from Bender Medsystems, Vienna, Austria) according to the protocol provided by the manufacturer.

### Statistical analysis

For between-group comparisons, the Kruskal-Wallis test, the Mann-Whitney *U*-test and Fisher's test were used, as appropriate. Correlations between continuous values were evaluated using Spearman's rank test. Potential relationships between individual parameters were assessed using univariate and multivariate regression analysis. All parameters with *P *<0.1 in univariate regression analysis were included in multivariate regression. For all other tests *P *<0.05 was considered significant. Values are given as mean and SD unless stated otherwise. Values in SSc patients higher than the mean + 2 SD of the control group were considered high. To assess the association between high concentrations of soluble proteins, or high sCD163/sTWEAK ratio and the presence of DU, the odds ratio (OR) with 95% CI was calculated.

## Results

### Clinical characteristics of SSc patients

Of the patients, 20 (23%) had dSSc and the remaining 69 (77%) had lSSc. HRCT of the lungs revealed ILD in 55 patients (62%). PH was found in 4 out of 81 (5%) patients in whom the results of echocardiography were available. Thirty-eight patients (43%) had DU at the time of the study. None of the patients had scleroderma renal crisis. ANA, anti-topo I antibodies and ACA were found in 84 (94%), 35 (39%) and 33 (37%) patients, respectively. Arterial hypertension of unknown cause (considered essential) was found in 8 SSc patients (9%) and none of the control subjects. When enrolled into the study, 27 SSc patients (30%) were being treated with calcium channel blockers (CCBs), 33 (37%) with pentoxyphylline, 12 (14%) with angiotensin-converting enzyme inhibitors (ACEIs), 4 (5%) with sildenafil and 13 (15%) with non-steroidal anti-inflammatory drugs (NSAIDs). Detailed clinical characteristics of the SSc patients are presented in Table 1.

### Serum concentrations of sCD163 and sTWEAK

**Figure 1:**
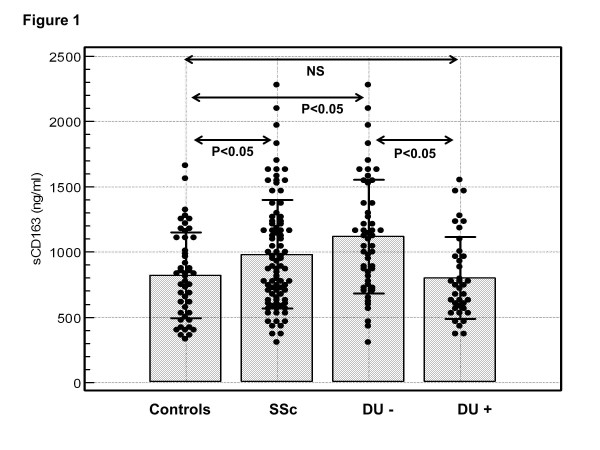
**Serum concentration of sCD163 in patients with systemic sclerosis (SSc) and healthy controls**. Concentration of sCD163 in the sera of all SSc patients (SSc), SSc patients without digital ulcers (DU-), SSc patients with digital ulcers (DU+) and healthy controls. Closed circles represent values in individual subjects, bars represent the mean values of the groups, and horizontal lines the appropriate SDs. NS, not significant.

The mean concentration of sCD163 in patients with SSc (mean ± SD 984 ± 420 ng/mL, range 302 to 2,275 ng/mL) was significantly higher than in HC (823 ± 331 ng/mL, range 329 to 1,659 ng/mL; *P *= 0.023) (Figure 1). High sCD163 concentration, defined as greater than the mean + 2 SD of the value in HC (1,485 ng/mL), was found in 13 SSc patients (15%). Comparison of those 13 SSc patients with high sCD163 concentration with the remaining 76 SSc patients revealed that patients with high serum sCD163 were less likely to have DU compared with those with normal sCD163 concentration (Table 2). Indeed, DU were present in only one out of thirteen (8%) SSc patients with high serum sCD163 concentration compared with 37 out of 76 (48%) SSc patients with normal sCD163 levels (OR 0.09, 95% CI 0.01, 0.71; *P *<0.05). Accordingly, the mean concentration of sCD163 in patients without DU (1,119 ± 439 ng/mL) was significantly higher than in patients with DU (802 ± 317 ng/mL, *P *= 0.002) (Figure 1). Moreover, the mean concentration of sCD163 in the former subgroup of SSc patients was significantly higher than in HC (*P *= 0.0008), whereas the mean concentration of sCD163 in SSc patients with DU did not differ significantly from the control group (*P *>0.05) (Figure 1).

**Table 2:** Comparison of demographic, clinical and laboratory features of SSc patients with high and normal concentration of sCD163 and high and normal sCD163/sTWEAK ratios.

Characteristic^a^	sCD163			sCD163/sTWEAK		
	
	High* (*n *= 13)	Normal (*n *= 76)	*P*-value	High* (*n *= 29)	Normal (*n *= 60)	*P*-value
Age, years	56.08 ± 16.11	57.42 ± 11.67	NS	59.38 ± 12.86	56.18 ± 12.01	NS
Disease duration, years	8.23 ± 6.91	11.66 ± 9.29	NS	11.53 ± 7.81	10.23 ± 9.55	NS
Duration of RP (data available for 54 patients), years	11.46 ± 8.04	9.11 ± 8.58	NS	10.36 ± 7.55	9.32 ± 8.82	NS
DSSc, n/total	5/13 (38.46%)	15/76 (19.74%)	NS	10/29 (34.48%)	10/60 (16.67%)	0.055
mRSS (data available for 72 patients)	10.92 ± 9.78	7.95 ± 6.58	NS	12.30 ± 9.68	6.63 ± 4.85	**0.0085**
ILD, n/total	9/13 (69.23%)	46/76 (60.53%)	NS	17/29 (58.6%)	38/60 (63.33%)	NS
PH, n/total (data available for 81 patients)	2/13 (15.39%)	2/68 (2.94%)	NS	2/29 (6.90%)	2/52 (3.85%)	NS
FVC (data available for 75 patients), % of predicted	86.33 ± 19.89	94.56 ± 21.61	NS	95.42 ± 23.40	92.37 ± 20.26	NS
DLCO (data available for 53 patients), % of predicted	74.38 ± 22.94	80.10 ± 30.32	NS	73.16 ± 24.73	82.19 ± 30.97	NS
PASP (data available for 56 patients), mmHg	32.86 ± 12.03	29.23 ± 10.99	NS	29.00 ± 9.25	30.13 ± 12.23	NS
Digital ulcers, n/total	1/13 (7.69%)	37/76 (46.68%)	**0.005**	5/29 (17%)	33/60 (55%)	**0.0006**
Anti-topo I, n/total	4/13 (30.77%)	31/76 (40.79%)	NS	10/29 (35%)	25/60 (41.67%)	NS
ACA, n/total	3/13 (23.08%)	30/76 (39.47%)	NS	9/29 45%)	24/60 (40%)	NS
ESR (data available for 51 patients), mm/h	28.18 ± 21.05	21.78 ± 19.27	NS	19.64 ± 11.47	24.49 ± 21.93	NS
Arterial hypertension, n/total	2/13 (15.39%)	6/76 (7.90%)	NS	3/29 (10.35%)	5/60 (8.33%)	NS
CCBs, n/total Pentoxyphylline, n/total ACEIs, n/total Sildenafil, n/total NSAIDs, n/total	3/13 (23.08%) 5/13 (38.46%) 4/13 (30.77%) 1/13 (7.69%) 3/13 (23.08%)	25/76 (32.90%) 29/76 (38.16%) 8/76 (10.53%) 3/76 (3.95%) 10/76 (13.16%)	NS NS NS NS NS	8/29 (27.58%) 7/29 (24.14%) 4/29 (13.79%) 1/29 (3.45%) 4/29 (13.79%)	19/60 (31.67%) 26/60 (43.33%) 8/60 (13.33%) 3/60 (5.00%) 9/60 (15.00%)	NS NS NS NS NS

Because high concentrations of sTWEAK were found in two SSc patients only, no reliable statistical comparisons between SSc patients with high and those with normal sTWEAK concentration could be performed. Values are given as mean ± SD unless stated otherwise. ^a^Data available for 89 patients unless stated otherwise. *The high concentration of sCD163 and high sCD163/sTWEAK ratio was defined as a value greater than the mean + 2 SD of the value in healthy controls. sTWEAK, soluble tumour necrosis factor-like weak inducer of apoptosis; NS, not significant (*P *>0.05); ACA, anticentromere antibodies; ACEIs, angiotensin-converting enzyme inhibitors; ANA, antinuclear antibodies; CCBs, calcium channel blockers; DLCO, diffusing capacity of the lungs for carbon monoxide; dSSc, diffuse systemic sclerosis; ESR, erythrocyte sedimentation rate; FVC, forced vital capacity; HRCT, high resolution computed tomography; ILD, scleroderma interstitial lung disease; mRSS, modified Rodnan skin score; NSAIDs, non-steroidal anti-inflammatory drugs; PASP, pulmonary artery systolic pressure; PH, pulmonary hypertension; RP, Reynaud's phenomenon.

No significant association or correlation was found between serum sCD163 concentration and any other clinical or laboratory factor (Tables 2 and 3). There were no significant differences in serum concentration of sCD163 between SSc patients receiving different therapies or between those with and without arterial hypertension (data not shown), nor any other significant associations between different treatments or the presence of arterial hypertension and sCD163 levels (Table 2).

**Table 3:** Correlations between sCD163, sTWEAK and sCD163/sTWEAK ratio and clinical and laboratory parameters of patients with systemic sclerosis.

Parameter (number of subjects)	*R *Spearman (*P*-value)		
	sCD163	sTWEAK	sCD163/sTWEAK
Disease duration (89)	-0.09 (*P *>0.05)	-0.32 *P *= 0.002	0.23 *P *= 0.03
Duration of Raynaud's phenomenon (54)	0.14 (*P *>0.05)	0.11 (*P *>0.05)	0.03 (*P *>0.05)
mRSS (82)	0.03 (*P *>0.05)	-0.08 (*P *>0.05)	0.13 (*P *>0.05)
FVC (75)	-0.06 (*P *>0.05)	-0.22 (*P *>0.05)	0.09 (*P *>0.05)
DLCO (53)	-0.09 (*P *>0.05)	-0.05 (*P *>0.05)	-0.02 (*P *>0.05)
PASP (57)	-0.14 (*P *>0.05)	-0.20 (*P *>0.05)	0.11 (*P *>0.05)
ESR (51)	0.15 (*P *>0.05)	0.07 (*P *>0.05)	0.04 (*P *>0.05)

sTWEAK, soluble tumour necrosis factor-like weak inducer of apoptosis; DLCO, diffusing capacity of the lungs for carbon monoxide; ESR, erythrocyte sedimentation rate; FVC, forced vital capacity; mRSS, modified Rodnan skin score; PASP, pulmonary artery systolic pressure.

**Figure 2:**
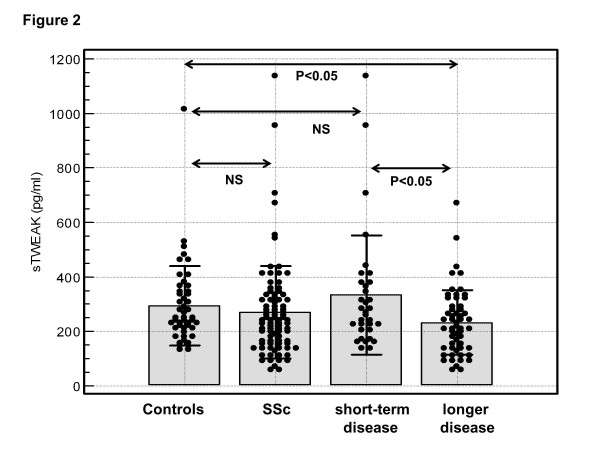
**Serum concentration of soluble tumour necrosis factor-like weak inducer of apoptosis (sTWEAK) in patients with systemic sclerosis (SSc) and controls**. Concentration of sTWEAK in the sera of all SSc patients (SSc), SSc patients with short-term disease (defined as not longer than 3 years in diffuse SSc and not longer than 5 years in limited SSc), those with longer disease duration and healthy controls. Closed circles represent values in individual subjects, bars represent the mean values of the groups, and horizontal lines the appropriate SDs. NS, not significant.

The mean serum concentrations of sTWEAK were comparable between patients with SSc (270 ± 171 pg/mL, range 56 to 1,135 pg/mL) and HC (294 ± 147 pg/mL, range 132 to 1,015 pg/mL, *P *>0.05) (Figure 2). In SSc patients sTWEAK showed a weak inverse correlation with duration of SSc (*R *= -0.32 *P *= 0.002) (Table 3). Accordingly, concentration of sTWEAK was significantly greater in 33 patients with short-term SSc (334 ± 222 pg/mL) as compared to the remaining 56 SSc patients with longer disease duration (232 ± 120 pg/mL, *P *= 0.01) (Figure 2). The mean concentration of sTWEAK in SSc patients with short-term disease did not differ significantly from the control group (*P *= 0.7) while sTWEAK levels in SSc patients with longer disease duration was significantly diminished compared with HC (*P *= 0.02). However, in multivariate regression analysis including disease duration and other clinical parameters of the disease, no significant association between sTWEAK and disease duration was found (data not shown).

High sTWEAK concentration (above the mean + 2SD of the control group, which was 588 pg/mL) was found in two patients only (Figure 2). Because of the small number of SSc patients with high sTWEAK levels, no reliable statistical comparisons between SSc patients with high or normal sTWEAK concentration were performed. There were no further significant associations between sTWEAK and clinical or laboratory parameters of the disease in SSc patients (Tables 2 and 3). There were no significant differences in serum concentration of sTWEAK between SSc patients receiving different therapies or between those with and without arterial hypertension (data not shown). Serum concentrations of sCD163 and sTWEAK did not correlate with each other either in patients with SSc (*R *= -0.05, *P *>0.05) or in the whole group (*R *= 0.1, *P *>0.05).

### sCD163/sTWEAK ratio and clinical and laboratory parameters of disease

**Figure 3:**
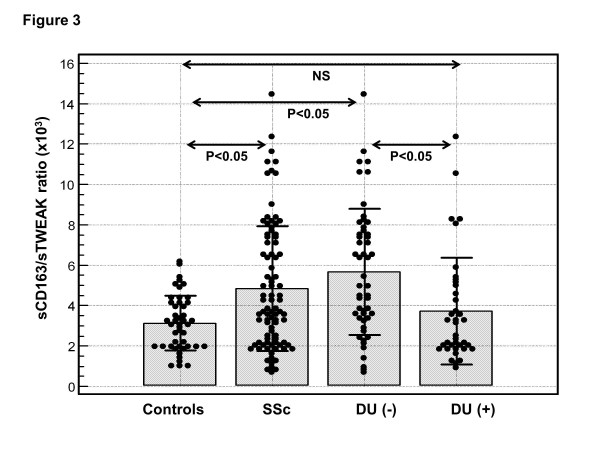
**sCD163/soluble tumour necrosis factor-like weak inducer of apoptosis (sTWEAK) in patients with systemic sclerosis (SSc) and healthy controls**. sCD163/sTWEAK ratio in the sera of all SSc patients (SSc), SSc patients without digital ulcers (DU-), SSc patients with digital ulcers (DU+) and healthy controls. Closed circles represent values in individual subjects, bars represent the mean values of the groups, and horizontal lines the appropriate SDs. NS, not significant.

The mean sCD163/sTWEAK ratio was significantly higher in patients with SSc (4,837 ± 3,103, range 660 to 14,440) compared with HC (3,115 ± 1,346, range 980 to 6,160; *P *<0.001) (Figure 3). High sCD163/sTWEAK ratio (greater than the mean + 2SD of HC, which was 5,807) was found in 29 (33%) SSc patients. Comparison of those 29 SSc patients with high sCD163/sTWEAK ratio to the remaining 60 SSc patients showed that high sCD163/sTWEAK ratio was associated with greater skin involvement as indicated by higher mRSS (Table 2). The diffuse form of the disease was twice as frequent (35%) in patients with high sCD163/sTWEAK ratio as in SSc patients with normal sCD163/sTWEAK ratio (18%), but the difference was of borderline significance (*P *= 0.055) (Table 2). Moreover, patients with high sCD163/sTWEAK ratio had lower risk of DU (Table 2): 5 out of 29 (17%) of the SSc patients with high sCD183/sTWEAK ratio had DU at the time of the study compared with 33 out of 60 (55%) of those with normal sCD183/sTWEAK ratio (OR 0.17, 95% CI 0.06, 0.51; *P *<0.05). The sCD163/sTWEAK ratio was significantly greater in patients without DU (5,664 ± 3,158) compared with patients with DU (3,727 ± 2,687, *P *= 0.001) (Figure 3). The sCD163/sTWEAK ratio in patients without DU was also significantly greater as compared with HC (*P *= 0.0001) (Figure 3).

In the whole group of SSc patients, sCD163/sTWEAK ratio showed a weak correlation with duration of the disease (*R *= 0.23, *P *= 0.03). However, the sCD163/sTWEAK ratio was not significantly different between patients with shorter disease duration (4,101 ± 2,650) compared with patients with longer duration of SSc (5,271 ± 3,287, *P *= 0.09).

In multivariate regression analysis, including mRSS, the presence of DU and disease duration as independent values, only mRSS (β coefficient = 0.35, *P *<0.01) and the presence of DU (β coefficient = -0.38, *P *<0.001) but not disease duration (β coefficient = 0.2, *P *>0.05) were independently associated with sCD163/sTWEAK ratio.

No significant association or correlation was found between the sCD163/sTWEAK ratio and other clinical or laboratory features of the disease (Tables 2 and 3). There were no significant differences in sCD163/sTWEAK ratio between SSc patients receiving different therapies or between those with and without arterial hypertension (data not shown), nor any other significant associations between treatments received by SSc patients or the presence of arterial hypertension and sCD163/sTWEAK ratio (Table 2).

**Figure 4:**
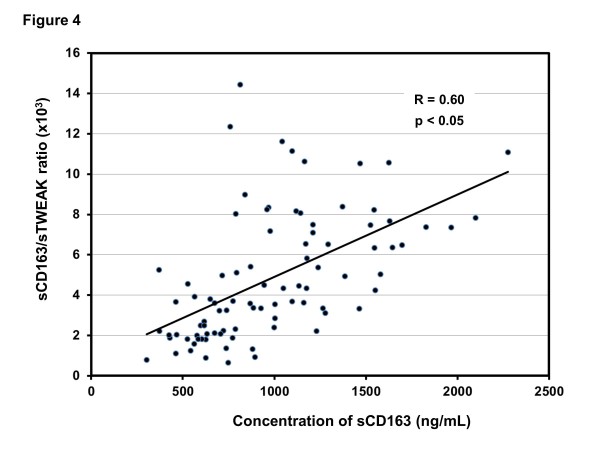
**Correlation between concentration of sCD163 and sCD163/soluble tumour necrosis factor-like weak inducer of apoptosis (sTWEAK) ratio in patients with systemic sclerosis (SSc)**.

Both high concentration of sCD163 and high sCD163/sTWEAK ratio were associated with lower risk of DU in SSc patients (Table 2). Analysis of individual SSc patients revealed that the majority of patients (11 out of 13, 85%) with high sCD163 levels also had high sCD163/sTWEAK ratio. In the remaining 2 of the 13 SSc patients with high sCD163 levels the sCD163/sTWEAK ratio was lower than 2 SD above the mean in the controls (due to relatively high levels of sTWEAK). Correlation between concentration of sCD163 and sCD163/sTWEAK ratio in individual SSc patients is shown in Figure 4.

DU were present in 5 out of 18 (28%) SSc patients with high sCD163/sTWEAK ratios, but at a normal sCD163 level, and in none of the SSc patients who had both high sCD163 levels and sCD163/sTWEAK ratios. Thus, in our study the presence of both high sCD163 level and high sCD163/sTWEAK ratio was a better predictor of DU in SSc patients than a high sCD163/sTWEAK ratio alone.

## Discussion

To the best of our knowledge this is the first study evaluating relationships between serum sCD163 and sTWEAK in patients with SSc. We show that the sCD163/sTWEAK ratio is significantly increased in patients with scleroderma compared with healthy subjects, and that a high sCD163/sTWEAK ratio is associated with greater skin involvement but lower risk of DU in SSc. Moreover, high serum concentration of sCD163 was independently associated with fewer DU, indicating that the processes that lead to elevated secretion of sCD163 and/or sCD163 itself might be involved in protection against development of DU in SSc.

In the present study the mean serum concentration of sCD163 in the whole population of SSc patients was significantly higher than in HC. Our results are in agreement with two recent reports by Nakayama *et al*. and Shimizu *et al*. In those two studies, which included 43 and 56 Japanese patients with SSc, respectively, serum concentration of sCD163 was significantly higher in scleroderma patients compared with the control group [23,24]. We have confirmed those observations in a greater population derived from several clinical centres.

CD163 is synthesized exclusively by monocytes/macrophages and originally expressed as a membrane protein, which is shed from the cell surface in a process that is dependent on proteolytic enzymes [4-6]. The shedding occurs constitutively but can also be induced by inflammatory stimuli or oxidative stress [36]. Because of the constant shedding of membrane CD163 it is assumed that the blood levels of sCD163 may reflect its overall synthesis by mononuclear phagocytes [37]. Indeed, Higashi-Kuwata *et al*. reported increased expression of CD163 on skin macrophages and a subset of peripheral blood mononuclear cells in SSc patients compared with healthy subjects, which is also in line with our present findings [38].

Interestingly, elevated serum sCD163 levels in our study were associated with a significantly lower risk of DU. Of note, was that previous studies had found an association between high serum sCD163 levels and greater frequency of PH or higher pulsatility index, which is a surrogate marker of kidney arterial injury [23,24]. However, neither of these two previous reports separately evaluated relationships between serum sCD163 levels and active DU [23,24]. Thus, our results and those of others indicate an association between CD163 and the involvement of blood vessels in SSc. The discrepancies between the studies concerning significant association with particular forms of vessel involvement might be attributed to the differences in the clinical profile of patients involved in different studies as well as to methodological differences in clinical assessments.

The results of our study do not allow us to make firm conclusions about the role of CD163 in the pathogenesis of DU in scleroderma. However, there are several mechanisms through which CD163 might protect against development of DU. As a scavenger receptor responsible for removal of haemoglobin-haptoglobin complexes, CD163 protects from the toxic effects of free haemoglobin and oxidative stress [39]. This function of CD163 might be of special interest in SSc because microvascular injury is a constant feature of the disease and might result in oxidative stress and increased injury to erythrocytes, with subsequent release of free haemoglobin [40]. Elevated expression of CD163 has been associated with triggering anti-inflammatory effects in response to extracellular haemoglobin [9,39]. This phenomenon might also be of relevance in SSc because inflammatory reaction, in addition to vascular disease, is believed to be responsible for increased oxidative stress in scleroderma patients [40]. It has also been shown that CD163-expressing cells possess pro-angiogenic activity and may stimulate formation of new blood vessels through activation of proliferation of endothelial cells [41]. The latter phenomenon might be directly involved in healing of DU and appears to be an appealing explanation of the association between high serum sCD163 concentration and absence of DU in SSc as observed in our study.

As discussed above, it has been shown that CD163 can bind and neutralize TWEAK, a newly identified member of the TNFα superfamily, which is involved in the regulation of inflammation, angiogenesis and connective tissue remodelling [16-21]. Although TWEAK is involved in the regulation of processes that are considered of key importance in the pathogenesis of SSc, so far only one study has investigated blood levels of sTWEAK in SSc [25]. Yanaba *et al*. found significantly higher levels of sTWEAK in sera from SSc patients compared with HC [25]. Unlike Yanaba *et al*., in the present study we did not find significant differences in the serum concentration of sTWEAK between SSc patients and the control group. The discrepancies between those two studies can be attributed to differences in the clinical characteristics of the patients. Moreover, our present findings are in agreement with our previous study showing that synthesis of sTWEAK by peripheral blood mononuclear cells is comparable between SSc patients and healthy subjects [42].

Consistent with significantly higher sCD163 concentration in SSc patients and lack of difference in sTWEAK levels between SSc patients and HC, in our study the sCD163/sTWEAK ratio was significantly increased in SSc patients compared with healthy subjects. Both high sCD163 and sCD163/sTWEAK ratio were associated with lower risk of DU, suggesting that elevated expression of CD163 plays a crucial role in that phenomenon, and the effects are not associated with its ability to neutralize TWEAK. However, in our study high sCD163/sTWEAK ratio, but not sCD163 alone, was associated with greater extent of skin fibrosis, indicating that the CD163-TWEAK interaction might be specifically involved in the progression of skin disease in SSc The significance of our findings remains to be established. At present little is known about the role of CD163 and TWEAK in the pathogenesis of fibrosis and the available evidence appears controversial. On one hand, CD163 positive macrophages, which are involved in wound healing, have been shown to exert profibrotic function [43]. Moreover, in the study by Yanaba *et al*., serum concentration of sTWEAK inversely correlated with the risk of ILD, indicating that TWEAK might have a protective role against the development of fibrosis in SSc patients [25]. On the other hand, the results of *in vitro *studies suggest that inhibition of TWEAK by CD163 might have a potential anti-fibrotic effect because, as discussed above, TWEAK can directly activate fibroblast functions [19-21]. Further studies are therefore required to explain the role of CD163-TWEAK interactions in the development of skin fibrosis in SSc.

## Conclusions

In conclusion, we showed that serum concentration of sCD163 is increased in SSc patients and that high serum sCD163 level and sCD163/sTWEAK ratio are associated with lower risk of DU in patients with scleroderma. Moreover, the results of our study indicate that interactions between CD163 and TWEAK might play a role in the development of skin fibrosis. This appears of great interest with respect to improvement of the management of patients with scleroderma. Evaluation of sCD163 and sCD163/sTWEAK ratio might be helpful in the assessment of the risk of DU in SSc. Further studies are required to reveal whether the targeting of the CD163-TWEAK pathway might be a potential strategy for treating vascular disease and/or skin fibrosis in SSc.

## Abbreviations

ACA: anticentromere antibodies; ACEI: angiotensin-converting enzyme inhibitor; ANA: antinuclear antibodies; anti-topo I: anti-topoisomerase antibodies; CCB: calcium channel blocker; DLCO: diffusion capacity of the lungs for carbon monoxide; DU: digital ulcers; dSSc: diffuse cutaneous systemic sclerosis; ELISA: enzyme-linked immunosorbent assay; ESR: erythrocyte sedimentation rate; FGF: fibroblast growth factor; FVC: forced vital capacity; HC: healthy controls; HRCT: high resolution computed tomography; ILD: interstitial lung disease; lSSc: limited cutaneous systemic sclerosis; mRSS: modified Rodnan skin score; NF-kB: nuclear factor-kB;

NS: not significant; NSAID: non-steroidal anti-inflammatory drug; OR: odds ratio; PASP: pulmonary artery systolic pressure; PH: pulmonary hypertension; sCD163: soluble CD163; sTWEAK: soluble tumour necrosis factor-like weak inducer of apoptosis; SSc: systemic sclerosis; TNF: tumour necrosis factor; TWEAK: tumour necrosis factor-like weak inducer of apoptosis; VEGF: vascular endothelial growth factor.

## Competing interests

The authors declare that they have no Competing interests.

## Authors' contributions

OKB participated in the development of the concept and design of the study, data acquisition, analysis and interpretation of the results, and manuscript writing. MBi participated in the development of the concept of the study, data acquisition, analysis and interpretation of the results, and manuscript writing. SG, BTB and MMJ participated in acquisition, analysis and interpretation of data. MMC participated in study design, acquisition, analysis and interpretation of data. MBr and DK participated in study design, acquisition, analysis and interpretation of data. LC participated in study design, data acquisition and analysis, and drafting the manuscript. KK conceived the study, participated in analysis and interpretation of the results and manuscript writing. All authors have critically revised the manuscript and approved its final version.

## References

[B1] Abraham DJ Krieg T Distler J Distler O Overview of pathogenesis of systemic sclerosis Rheumatology (Oxford) 2009 15 Suppl 3 iii3 7 19487220 10.1093/rheumatology/ken481

[B2] Lafyatis R York M Innate immunity and inflammation in systemic sclerosis Curr Opin Rheumatol 2009 15 617 622 10.1097/BOR.0b013e32832fd69e 19633559 PMC2848972

[B3] Law SKA Micklem KJ Shaw JM Zhang XP Dong Y Willis AC Mason DY A new macrophage differentiation antigen which is a member of the scavenger receptor superfamily Eur J Immunol 1993 15 2320 2325 10.1002/eji.1830230940 8370408

[B4] Kristiansen M Graversen JH Jacobsen C Sonne O Hoffman HJ Law SK Moestrup SK Identification of the haemoglobin scavenger receptor Nature 2001 15 198 201 10.1038/35051594 11196644

[B5] Hintz KA Rassias AJ Wardwell K Moss ML Morganelli PM Pioli PA Givan AL Wallace PK Yeager MP Guyre PM Endotoxin induces rapid metalloproteinase-mediated shedding followed by up-regulation of the monocyte hemoglobin scavenger receptor CD163 J Leukoc Biol 2002 15 711 717 12377940

[B6] Etzerodt A Maniecki MB Moller K Moller HJ Moestrup SK Tumor necrosis factor alpha-converting enzyme (TACE/ADAM17) mediates ectodomain shedding of the scavenger receptor CD163 J Leukoc Biol 2010 15 1201 1205 10.1189/jlb.0410235 20807704

[B7] Matsushita N Kashiwagi M Wait R Nagayoshi R Nakamura M Matsuda T Hogger P Guyre PM Nagase H Matsuyama T Elevated levels of soluble CD163 in sera and fluids from rheumatoid arthritis patients and inhibition of the shedding of CD163 by TIMP-3 Clin Exp Immunol 2002 15 156 161 10.1046/j.1365-2249.2002.01963.x 12296867 PMC1906487

[B8] Kowal K Silver R Sławińska E Bielecki M Chyczewski L Kowal-Bielecka O CD163 and its role in inflammation Folia Histochem Cytobiol 2011 15 365 374 22038213 10.5603/fhc.2011.0052

[B9] Philippidis P Mason JC Evans BJ Nadra I Taylor KM Haskard DO Landis RC Hemoglobin scavenger receptor CD163 mediates interleukin-10 release and heme oxygenase-1 synthesis: anti-inflammatory monocyte-macrophage responses in vitro, in resolving skin blisters in vivo, and after cardiopulmonary bypass surgery Circ Res 2004 15 119 126 10.1161/01.RES.0000109414.78907.F9 14656926

[B10] Hogger P Sorg C Soluble CD163 inhibits phorbol ester-induced lymphocyte proliferation Biochem Biophys Res Commun 2001 15 841 843 10.1006/bbrc.2001.5845 11688984

[B11] Frings W Dreier J Sorg C Only soluble form of the scavenger receptor CD163 acts inhibitory on phorbol ester-activated T-lymphocytes, whereas membrane-bound protein has no effect FEBS Letters 2002 15 93 96 10.1016/S0014-5793(02)03142-3 12208511

[B12] Kowal K Moller HJ DuBuske LM Moestrup SK Bodzenta-Łukaszyk A Differential expression of monocyte CD 163 in single - and dual-asthmatic responders during allergen-induced bronchoconstriction Clin Exp Allergy 2006 15 1584 1591 10.1111/j.1365-2222.2006.02573.x 17177682

[B13] Topoll HH Zwadlo G Lange DE Sorg C Phenotypic dynamics of macrophage subpopulations during experimental gingivitis J Peridont Res 1989 15 106 112 10.1111/j.1600-0765.1989.tb00864.x 2524575

[B14] Zwadlo G Voegeli R Osthoff K Sorg C A mAb to a novel differentiation antigen on human macrophages associated with the down-regulatory phase of the inflammatory process Exp Cell Biol 1987 15 295 304 3450546 10.1159/000163432

[B15] Bover LC Cardó-Vila M Kuniyasu A Sun J Rangel R Takeya M Aggarwal BB Arap W Pasqualini R A previously unrecognized protein-protein interaction between TWEAK and CD163: potential biological implications J Immunol 2007 15 8183 8194 17548657 10.4049/jimmunol.178.12.8183

[B16] Winkles JA The TWEAK-Fn14 cytokine-receptor axis: discovery, biology and therapeutic targeting Nat Rev Drug Discov 2008 15 411 425 10.1038/nrd2488 18404150 PMC3018765

[B17] Zheng TS Burkly LC No end in site: TWEAK/Fn14 activation and autoimmunity associated- end-organ pathologies J Leukoc Biol 2008 15 338 347 10.1189/jlb.0308165 18483204

[B18] Kim SH Kang YJ Kim WJ Woo DK Lee Y Kim DI Park YB Kwon BS Park JE Lee WH TWEAK can induce pro-inflammatory cytokines and matrix metalloproteinase-9 in macrophages Circ J 2004 15 396 399 10.1253/circj.68.396 15056843

[B19] Chicheportiche Y Chicheportiche R Sizing I Thompson J Benjamin CB Ambrose C Dayer JM Proinflammatory activity of TWEAK on human dermal fibroblasts and synoviocytes: blocking and enhancing effects of anti-TWEAK monoclonal antibodies Arthritis Res 2002 15 126 133 10.1186/ar388 11879548 PMC83846

[B20] Ebihara N Nakayama M Tokura T Ushio H Murakami A Expression and function of fibroblast growth factor-inducible14 in human corneal myofibroblasts Exp Eye Res 2009 15 256 262 10.1016/j.exer.2009.03.014 19344712

[B21] Hosokawa Y Hosokawa I Ozaki K Nakae H Matsuo T Proinflammatory effects of tumour necrosis factor-like weak inducer of apoptosis (TWEAK) on human gingival fibroblasts Clin Exp Immuno 2006 15 540 549 10.1111/j.1365-2249.2006.03233.x PMC1810398 17100776

[B22] Moreno JA Muñoz-García B Martín-Ventura JL Madrigal-Matute J Orbe J Páramo JA Ortega L Egido J Blanco-Colio LM The CD163-expressing macrophages recognize and internalize TWEAK: potential consequences in atherosclerosis Atherosclerosis 2009 15 103 110 10.1016/j.atherosclerosis.2009.04.033 19473660

[B23] Nakayama W Jinnin M Makino K Kajihara I Makino T Fukushima S Inoue Y Ihn H Serum levels of soluble CD163 in patients with systemic sclerosis Rheumatol Int 2012 15 403 407 10.1007/s00296-010-1691-z 21120485

[B24] Shimizu K Ogawa F Yoshizaki A Akiyama Y Kuwatsuka Y Okazaki S Tomita H Takenaka M Sato S Increased serum levels of soluble CD163 in patients with scleroderma Clin Rheumatol 2012 15 1059 1064 10.1007/s10067-012-1972-x 22453843

[B25] Yanaba K Yoshizaki A Muroi E Hara T Ogawa F Usui A Hasegawa M Fujimoto M Takehara K Sato S Elevated circulating TWEAK levels in systemic sclerosis: association with lower frequency of pulmonary fibrosis J Rheumatol 2009 15 1657 1662 10.3899/jrheum.081310 19531747

[B26] Subcommittee for scleroderma criteria of the American Rheumatism Association Diagnostic and Therapeutic Criteria Committee Preliminary criteria for the classification of systemic sclerosis (scleroderma) Arthritis Rheum 1980 15 581 590 10.1002/art.1780230510 7378088

[B27] LeRoy EC Medsger TA Jr Criteria for the classification of early systemic sclerosis J Rheumatol 2001 15 1573 1576 11469464

[B28] LeRoy EC Black C Fleischmajer R Jablonska S Krieg T Medsger TA Jr Rowell N Wollheim F Scleroderma (systemic sclerosis): classification, subsets and pathogenesis J Rheumatol 1988 15 202 205 3361530

[B29] Medsger TA Jr Natural history of systemic sclerosis and the assessment of disease activity, severity, functional status, and psychologic well-being Rheum Dis Clin North Am 2003 15 255 273 10.1016/S0889-857X(03)00023-1 12841294

[B30] Postlethwaite AE Wong WK Clements P Chatterjee S Fessler BJ Kang AH Korn J Mayes M Merkel PA Molitor JA Moreland L Rothfield N Simms RW Smith EA Spiera R Steen V Warrington K White B Wigley F Furst DE A multicenter, randomized, double-blind, placebo-controlled trial of oral type I collagen treatment in patients with diffuse cutaneous systemic sclerosis: I. oral type I collagen does not improve skin in all patients, but may improve skin in late-phase disease Arthritis Rheum 2008 15 1810 1822 10.1002/art.23501 18512816 PMC4511098

[B31] Muangchan C Harding S Khimdas S Bonner A Canadian Scleroderma Research group Baron M Pope J Association of C-reactive protein with high disease activity in systemic sclerosis: Results from the Canadian Scleroderma Research Group Arthritis Care Res (Hoboken) 2012 15 1405 1414 10.1002/acr.21716 22556030

[B32] Clements PJ Hurwitz EL Wong WK Seibold JR Mayes M White B Wigley F Weisman M Barr W Moreland L Medsger TA Jr Steen VD Martin RW Collier D Weinstein A Lally E Varga J Weiner SR Andrews B Abeles M Furst DE Skin thickness score as a predictor and correlate of outcome in systemic sclerosis: high-dose versus low-dose penicillamine trial Arthritis Rheum 2000 15 2445 2454 10.1002/1529-0131(200011)43:11<2445::AID-ANR11>3.0.CO;2-Q 11083267

[B33] Mukerjee D St George D Knight C Davar J Wells AU Du Bois RM Black CM Coghlan JG Echocardiography and pulmonary function as screening tests for pulmonary arterial hypertension in systemic sclerosis Rheumatology (Oxford) 2004 15 461 466 10.1093/rheumatology/keh067 15024134

[B34] Amanzi L Braschi F Fiori G Galluccio F Miniati I Guiducci S Conforti ML Kaloudi O Nacci F Sacu O Candelieri A Pignone A Rasero L Conforti D Matucci-Cerinic M Digital ulcers in scleroderma: staging, characteristics and sub-setting through observation of 1614 digital lesions Rheumatology (Oxford) 2010 15 1374 1382 10.1093/rheumatology/keq097 20400463

[B35] Beyer C Distler JH Allanore Y Aringer M Avouac J Czirják L Cutolo M Damjanov N Del Galdo F Fligelstone K Guiducci S Kowal-Bielecka O van Laar JM Martucci-Cerinic M Müller-Ladner U Riemekasten G Tarner IH Tyndall A Kennedy AT Valentini G Vettori S Walker UA Denton C Distler O EUSTAR Biobanking Group EUSTAR biobanking: recommendations for the collection, storage and distribution of biospecimens in scleroderma research Ann Rheum Dis 2011 15 1178 1182 10.1136/ard.2010.142489 21285118

[B36] Buechler C Ritter M Orso E Langmann T Klucken J Schmitz G Regulation of scavenger receptor CD163 expression in human monocytes and macrophages by pro- and anti-inflammatory stimuli J Leukoc Biol 2000 15 97 103 10648003

[B37] Droste A Sorg C Högger P Shedding of CD163, a novel regulatory mechanism for a member of the scavenger receptor cysteine-rich family Biochem Biophys Res Communic 1999 15 110 10.1006/bbrc.1999.0294 10066432

[B38] Higashi-Kuwata N Jinnin M Makino T Fukushima S Inoue Y Muchemwa FC Yonemura Y Komohara Y Takeya M Mitsuya H Ihn H Characterization of monocyte/macrophage subsets in the skin and peripheral blood derived from patients with systemic sclerosis Arthritis Res Ther 2010 15 R128 10.1186/ar3066 20602758 PMC2945018

[B39] Martin-Ventura JL Madrigal-Matute J Martinez-Pinna R Ramos-Mozo P Blanco-Colio LM Moreno JA Tarin C Burillo E Fernandez-Garcia CE Egido J Meilhac O Michel JB Erythrocytes, leukocytes and platelets as a source of oxidative stress in chronic vascular diseases: Detoxifying mechanisms and potential therapeutic options Thromb Haemost 2012 15 435 442 10.1160/TH12-04-0248 22836558

[B40] Gabrielli A Svegliati S Moroncini G Amico D New insights into the role of oxidative stress in scleroderma fibrosis Open Rheumatol J 2012 15 87 95 10.2174/1874312901206010087 22802906 PMC3395898

[B41] Mayer A Hiebl B Lendlein A Jung F Support of HUVEC proliferation by pro-angiogenic intermediate CD163+ monocytes/macrophages: a co-culture experiment Clin Hemorheol Microcirc 2011 15 423 430 22214713 10.3233/CH-2011-1492

[B42] Bielecki M Kowal K Lapinska A Chwiecko J Skowronski J Sierakowski S Chyczewski L Kowal-Bielecka O Diminished production of TWEAK by the peripheral blood mononuclear cells is associated with vascular involvement in patients with systemic sclerosis Folia Histochem Cytobiol 2009 15 465 469 20164033 10.2478/v10042-009-0103-2

[B43] Juniantito V Izawa T Yamamoto E Murai F Kuwamura M Yamate J Heterogeneity of macrophage populations and expression of galectin-3 in cutaneous wound healing in rats J Comp Pathol 2011 15 378 389 10.1016/j.jcpa.2011.01.012 21435650

